# Comparative plastid genomics of *Hippophae* reveals phylogenetic relationships and provides candidate DNA markers for taxonomic identification

**DOI:** 10.1038/s41598-026-40776-0

**Published:** 2026-03-15

**Authors:** Nobuaki Asakura, Masato Noda, Yusei Takahashi, Shinji Ueno, Naoki Arai

**Affiliations:** 1 Department of Biochemistry and Biotechnology, Faculty of Chemistry and Biochemistry, Kanagawa University, Rokkakubashi 3-27-1, Kanagawa-ku, Yokohama, Japan; 2 Department of Material and Life Chemistry, Faculty of Engineering, Kanagawa University, Rokkakubashi 3-27-1, Kanagawa-ku, Yokohama, Japan

**Keywords:** *Hippophae*, plastid genome, molecular phylogenetics, genetic diversity, nucleotide diversity, Evolution, Genetics, Molecular biology, Plant sciences

## Abstract

Sea buckthorn (*Hippophae* L., Elaeagnaceae) is of considerable ecological and economic importance, and primarily distributed across the Qinghai–Tibet Plateau and adjacent regions. Morphological similarity among taxa has long hindered accurate species and subspecies identification, underscoring the need for robust molecular diagnostics. This study analyzed the complete plastid genome sequences of five *Hippophae* species (17 accessions), including a newly assembled genome of *Hippophae rhamnoides* subsp. *mongolica*. The genomes (~ 155–156 kb) exhibited a conserved quadripartite structure comprising 85 protein-coding, eight rRNA, and 38 tRNA genes. Phylogenomic reconstruction based on 78 protein-coding genes well-resolved the interspecific relationships, confirmed the monophyly of *Hippophae* and *H. rhamnoides*, and consistently placed *H. tibetana* within the *H. rhamnoides* clade. Comparative analyses identified 46 highly variable regions and abundant A/T-rich simple sequence repeats, predominantly in intergenic spacers. Inverted repeat boundaries were largely conserved across taxa, with *H. salicifolia* exhibiting a distinctive *ndhF*–IRb/SSC configuration. These plastid genomic resources provide a robust foundation for the development of diagnostic molecular markers with direct applications in *Hippophae* taxonomy, phylogenetics, germplasm conservation, and targeted breeding programs.

**Supplementary Information:**

The online version contains supplementary material available at 10.1038/s41598-026-40776-0.

## Introduction

Sea buckthorn (*Hippophae* L., Elaeagnaceae) is a deciduous shrub or small tree (2–5 m in height, occasionally > 10 m) that is wind-pollinated and dioecious^1,2^, and sex determination is thought to be genetically controlled through an X/Y chromosome system, with males being heteromorphic^3–5^. The family Elaeagnaceae includes only two other genera, *Elaeagnus* and *Shepherdia*. Sea buckthorn is an economically and ecologically valuable plant that has been used for thousands of years in the Qinghai–Tibet Plateau (QTP) and surrounding regions^1,6–8^. It is highly tolerant to environmental stresses, such as extreme temperature, drought, high-altitude conditions, salinity, alkalinity, and inundation, with vigorous vegetative reproduction and a strong complex root system^1,6,7,9,10^. It can grow in nutritionally poor environments due to its nitrogen-fixing ability, making it useful for environmental conservation and ecological restoration, particularly soil erosion prevention, wind protection, land reclamation, and water conservation^6–10^. Its nutraceutical and medicinal importance are increasingly recognized owing to societal shifts toward healthy lifestyle and food habits^11,12^. The berries, leaves, and seeds are known for their high nutrient and bioactive contents, including vitamins (A, C, B1, B2, E, and K), flavonoids (isorhamnetin, quercetin, kaempferol, and rhamnetin), mineral elements (Ca, P, Fe, and K), oil, and other antioxidants^10–14^.

Formerly, the genus *Hippophae* was divided into three species—*H. rhamnoides*, *H. salicifolia*, and *H. tibetana*—and *H. rhamnoides* was subdivided into nine subspecies^15^. Taxonomic classification subsequently refined the genus to seven species—*H. rhamnoides*, *H. salicifolia*, *H. gyantsensis*, *H. goniocarpa*, *H. litangensis*, *H. neurocarpa*, and *H. tibetana*—with *H. rhamnoides* comprising eight subspecies including *carpatica*, *caucasica*, *fluviatilis*, *mongolica*, *rhamnoides*, *sinensis*, *turkestanica*, and *yunnanensis*^16^. Finally, the subsp. *wolongensis* was characterized^17^. All species are restricted to the Qinghai–Tibet Plateau and adjacent areas, except for common sea buckthorn, *H. rhamnoides*^8,15–18^. Natural *H. rhamnoides* populations are widely but discontinuously distributed from Asia to Europe, across 38 countries^15,19^. Domestication of common sea buckthorn began in Siberia in the 1930s, and its cultivation soon expanded to other regions of Russia and neighboring countries^20^. It has also been introduced to South and North America and more recently to Japan^6,21–24^.

Plant genetic resources (PGRs), such as landraces, wild relatives, and breeding lines, provide valuable genes for disease resistance and environmental adaptation and are crucial for sustainable and competitive plant breeding, providing the genetic diversity necessary for developing crop varieties with improved traits^25–27^. The conservation and expansion of PGRs are essential for maintaining biodiversity and ensuring food security^28^. In *Hippophae*, taxonomic classification of species or subspecies based on morphological traits is challenging due to their high similarity^16,29,30^. Taxon-specific molecular markers are required for the accurate classification of *Hippophae*, expansion of PGRs, and development of effective breeding programs.

Plastid genome sequences have proven valuable for developing molecular markers in various plant species. Chloroplast genomes have been used for phylogenetic analysis and cultivar identification in rice^31^, Chinese yam^32^, dandelion^33^, peanut^34^, and potato^35^, based on variation in single-nucleotide polymorphisms, simple sequence repeats (SSRs), and indels. Analyses of chloroplast genomes have identified variable regions that may serve as molecular markers. With advances in resources and technology, a considerable number of plastid genome sequences have been determined. Since the adoption of next-generation sequencing (NGS) in eudicot angiosperm research (especially *Nandina domestica* and *Platanus occidentalis*)^36^, plastid genomics has been widely implemented and greatly advanced^37,38^. In Elaeagnaceae, the first complete plastid genome sequence was determined for *Elaeagnus macrophylla*^39^, and that of *H. rhamnoides* was the first to be determined in *Hippophae*^40^. More recently, comparative chloroplast genome analyses in *Hippophae* have provided additional plastid genome resources, including assessments of plastid genome variation, identification of hypervariable regions and SSR loci, and plastid genome-based phylogenetic inference^41,42^.

In contrast to several previous plastid genome studies that focused primarily on limited species- or subspecies-level comparisons, we aimed to characterize the complete plastid genome of *H. rhamnoides* subsp. *mongolica* cv. Prevoskhodnaya and to reevaluate gene annotations and inverted repeat (IR) regions across 16 available *Hippophae *plastid genomes. Based on verified data, we: (1) compared the quadripartite structure, characteristics, gene content, and codon usage bias of 17 *Hippophae* plastid genomes to obtain baseline information; (2) constructed phylogenetic trees based on protein-coding genes (PCGs) to determine the applicability of plastid genome sequences in *Hippophae* systematics and taxonomy; (3) compared the nucleotide contents and IR boundary structures among the five *Hippophae* species and six *H. rhamnoides* subspecies, screening highly variable regions that may serve as molecular markers for species and subspecies identification; and (4) identified SSRs and their distribution across the 17 *Hippophae* plastid genomes and evaluated their potential as molecular markers.

## Methods

### DNA isolation, polymerase chain reaction (PCR) amplification, and sequencing

Leaf samples of *Hippophae rhamnoides* subsp. *mongolica* cv. Prevoskhodnaya were kindly provided by Prof. Yoshitaka Kawai, who cultivated the plants at the Atsugi Campus (Atsugi City, Kanagawa Prefecture) of the Faculty of Agriculture, Tokyo University of Agriculture, Japan. Total DNA was isolated from leaves using a DNeasy Plant Maxi Kit (Qiagen, Hilden, Germany) according to the manufacturer’s instructions. DNA concentration and purity were assessed using a NanoDrop One spectrophotometer (Thermo Fisher Scientific, Wilmington, DE, USA). DNA purity was evaluated using the A260/A280 ratio. To obtain DNA fragments covering the entire plastid genome, long-range PCR was performed using 14 primer sets (Table S1) designed using Primer3^43^. Each reaction mixture (50 µL) contained 1.25 units of PrimeSTAR GXL DNA Polymerase (Takara Bio, Otsu, Japan), 1× PrimeSTAR GXL Buffer (Takara Bio), dNTP mix (200 µ*M* each; Takara Bio), primers (250 n*M* each), and 50 ng template DNA. Thermal cycling conditions for PCR amplification were optimized depending on the DNA regions of the target sequences (Tables S2 and S3). The 14 PCR amplicons were divided into two pools (S1 and S2 described in Table S1) to determine both IR sequences independently. Plastid genome sequencing was performed by Hokkaido System Science Co. Ltd. (Hokkaido, Japan). After DNA fragmentation, sequencing libraries were prepared using a NEBNext Ultra II DNA Library Prep Kit (New England Biolabs, Ipswich, MA, USA). Paired-end sequencing (2 × 300 bp) was conducted on a MiSeq sequencing platform (Illumina, Canton, MA, USA). Adapter sequences (Read1: AGATCGGAAGAGCACACGTCTGAACTCCAGTCAC; Read2: AGATCGGAAGAGCGTCGTGTAGGGAAAGAGTGT) were removed using Cutadapt v1.1^44^ with the options “--match-read-wildcards”, “-O 1”, and “-a”. Because paired-end trimming was not supported in this version, Read1 and Read2 were processed separately, and read pairs containing ambiguous bases (N) in either read were filtered out using an awk script. Quality trimming was subsequently performed in paired-end mode using Trimmomatic v0.32^45^ with the following parameters: -phred33 LEADING:0 TRAILING:0 SLIDINGWINDOW:20:20 MINLEN:50. Adapter clipping (ILLUMINACLIP) was not applied. Unpaired reads were discarded, and only properly paired reads were retained for downstream analyses.

### Genome assembly, annotation, and codon usage

*De novo* assembly was performed using Velvet v1.2.10^46^. Velvet assemblies were generated using hash lengths (k-mer sizes) of 235 and 245, and velvetg was run with “-exp_cov auto”; all other parameters were kept as default. In addition, assembly was conducted using Platanus v1.2.4^47^ with default settings. Genome annotation was conducted with the GeSeq tool on ChloroBox-MPI-MPIPZ^48^, and a circular plastid gene map was generated using OrganellarGenomeDRAW^49^. Codon usage and relative synonymous codon usage (RSCU) were analyzed using CodonW v1.4.4 via the Galaxy platform (provided by the Institut Pasteur, https://galaxy.pasteur.fr/). In total, 78 PCGs were included in the analysis.

## Phylogenetic analysis

Phylogenetic analysis was conducted using the nucleotide sequences of 78 PCGs (*psbA*, *matK*, *rps16*, *psbK*, *psbI*, *atpA*, *atpF*, *atpH*, *atpI*, *rps2*, *rpoC2*, *rpoC1*, *rpoB*, *petN*, *psbM*, *psbD*, *psbC*, *psbZ*, *rps14*, *psaB*, *psaA*, *ycf3*, *rps4*, *ndhJ*, *ndhK*, *ndhC*, *atpE*, *atpB*, *rbcL*, *accD*, *psaI*, *ycf4*, *cemA*, *petA*, *psbJ*, *psbL*, *psbF*, *psbE*, *petL*, *petG*, *psaJ*, *rpl33*, *rps18*, *rpl20*, *rps12*, *clpP*, *psbB*, *psbT*, *psbN*, *psbH*, *petB*, *petD*, *rpoA*, *rps11*, *rpl36*, *rps8*, *rpl14*, *rpl16*, *rps3*, *rpl22*, *rps19*, *rpl2*, *rpl23*, *ycf2*, *ndhB*, *rps7*, *ycf1*, *ndhF*, *rpl32*, *ccsA*, *ndhD*, *psaC*, *ndhE*, *ndhG*, *ndhI*, *ndhA*, *ndhH*, and *rps15*) from the complete plastid genomes of 26 rosid species (ingroup), including five *Hippophae* and 10 *Elaeagnus* species (Table S4). *Nicotiana tabacum* was designated as the outgroup, bringing the total to 27 taxa. A second phylogenetic analysis was performed among the 17 complete plastid genomes of *Hippophae*, including the newly-sequenced genome in this study, using *Morus indica* as the outgroup. Multiple sequence alignment was conducted using MAFFT v7^50^. Maximum likelihood (ML) analysis was conducted with raxmlGUI v2.0.10^51^ using 1,000 bootstrap replicates after selecting the best-fit substitution model with ModelTest-NG^52^. The phylogenetic tree was visualized with FigTree v1.4.4 (https://github.com/rambaut/figtree/releases/tag/v1.4.4).

### Comparison of whole genome sequences and IR boundary regions

The complete plastid genomes were compared using mVISTA^53^ with the LAGAN alignment algorithm under default settings. The ML phylogenetic tree was used as a guide tree for sequence comparison. Nucleotide diversity within plastid genomes was estimated using a sliding window analysis with DnaSP v6.12.03^54^, with a window length of 600 bp and step size of 200 bp. We manually reevaluated the IR borders of the complete plastid genomes of known *Hippophae* species downloaded from the GenBank database (NCBI). To confirm the IR boundaries, we extracted sequences flanking the initially annotated IR regions and performed pairwise alignments using ClustalW^55^. The IR/LSC and IR/SSC junctions were then manually inspected based on the alignment results to verify and, where necessary, refine the IR border positions.

### Simple sequence repeat analysis

SSRs were identified using the online MISA-web tool (v2.1; https://webblast.ipk-gatersleben.de/misa/)^56,57^. SSR search parameters were set as: ten repeat units for mononucleotide; five repeat units for dinucleotide; four repeat units for trinucleotide; and three repeat units for tetra-, penta-, and hexanucleotide SSRs.

## Results

### Plastid genome organization

**Fig. 1:**
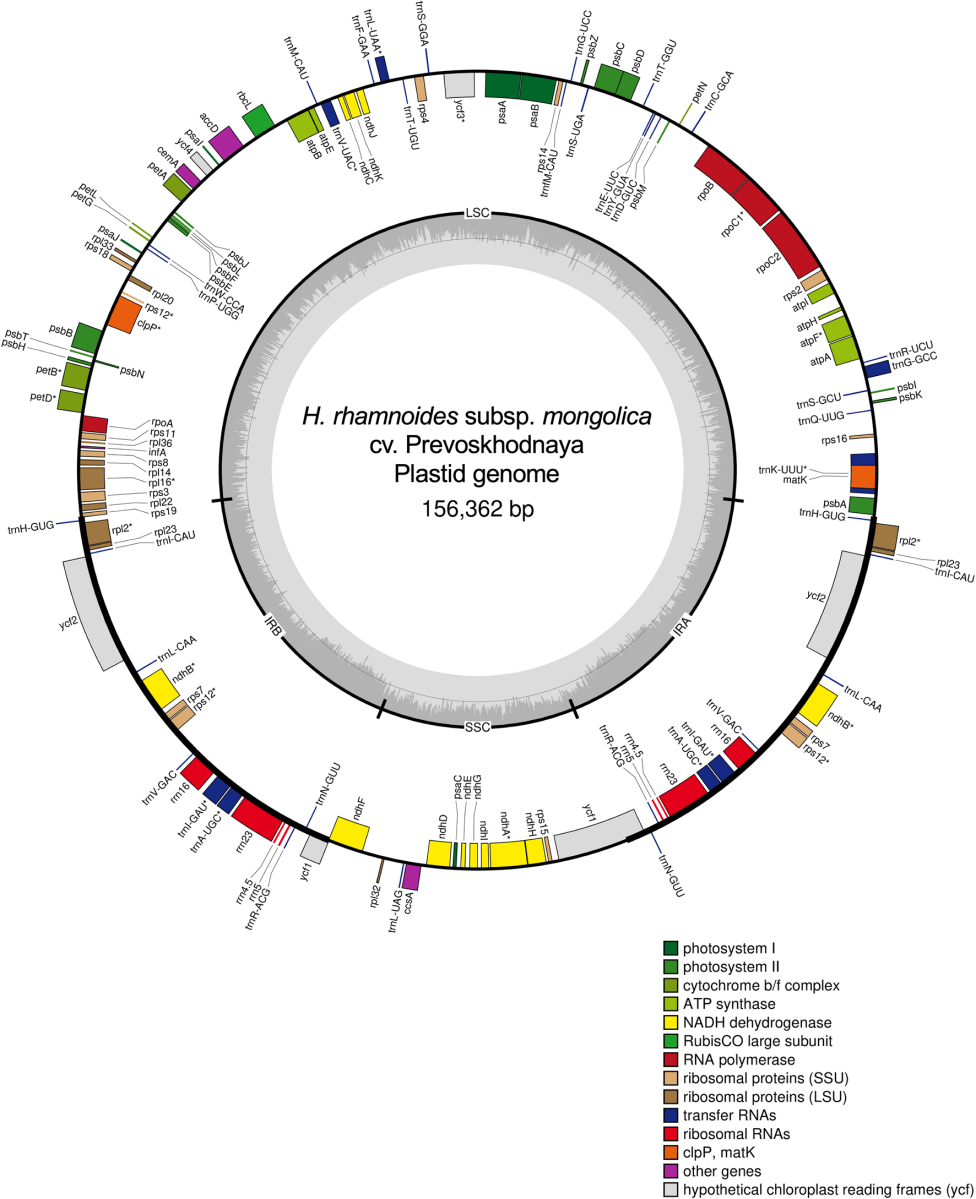
Physical map of complete plastid genome of *H. rhamnoides* subsp. *mongolica* cv. Prevoskhodnaya. Genes located outside and inside the outer circle are transcribed in the counterclockwise and clockwise directions, respectively. Color codes represent different functional gene groups. Inside the middle circle, GC and AT content variations are indicated by darker and lighter gray, respectively. * Genes containing introns are indicated.

The complete plastid genomes of five *Hippophae* species (17 accessions) ranged from 154,944 bp (*H. neurocarpa*, MW791512) to 156,415 bp (*H. rhamnoides* subsp. *yunnanensis*, NC_044479; Table 1). All plastid genomes exhibited the typical quadripartite structure, comprising LSC and SSC regions separated by two IR regions (Table 1; Fig. 1). The IR lengths of *H. tibetana* (MT512454), *H. gyantsensis* (NC_044478), *H. neurocarpa* (MT512453 and MW791512), and *H. salicifolia* (MW392804) were 2-, 3-, 3-, 3-, and 127-bp longer, respectively, than those recorded in the original database entries (Table 1).

**Table 1:** Comparison of complete plastid genomes in *Hippophae.*

Taxon		Accession no.	Total		LSC		SSC		IR	
			Length (bp)	GC (%)	Length (bp)	GC (%)	Length (bp)	GC (%)	Length (bp)	GC (%)
*H. rhamnoides*	subsp. *mongolica*	OM776960	156,362	36.6	84,001	34.5	19,043	29.8	26,659	42.4
	subsp. *mongolica*	MT512449	156,361	36.6	84,000	34.5	19,043	29.8	26,659	42.4
	subsp. *mongolica*	ON584762	156,310	36.6	83,966	34.5	19,036	29.9	26,654	42.4
	subsp. *sinensis*	NC_049156	156,355	36.6	84,002	34.5	19,037	29.8	26,658	42.4
	subsp. *yunnanensis*	NC_044479	156,415	36.7	84,072	34.6	19,047	30.0	26,648	42.4
	unknown subsp.	NC_035548	156,132	36.7	83,985	34.5	18,831	29.9	26,658	42.4
	subsp. *turkestanica*	MT512451	156,065	36.6	83,721	34.5	19,032	29.8	26,656	42.4
	subsp. *caucasica*	MT512452	156,185	36.6	83,842	34.5	19,025	29.8	26,659	42.4
	subsp. *rhamnoides*	MT512450	156,111	36.7	83,741	34.5	19,030	29.9	26,670	42.4
*H. tibetana*		MN643620	155,810	36.7	83,462	34.6	19,022	29.9	26,663	42.4
		MT512454	155,804	36.7	83,456	34.6	19,022	29.9	26,663	42.4
*H. gyantsensis*		NC_044478	155,260	36.7	83,022	34.6	18,894	30.0	26,672	42.4
*H. neurocarpa*	subsp. *neurocarpa*	MT512453	154,995	36.7	83,346	34.5	18,593	29.9	26,528	42.4
	unknown subsp.	MW791512	154,944	36.7	83,302	34.5	18,586	29.9	26,528	42.4
	unknown subsp.	NC_047483	156,316	36.6	83,968	34.5	19,042	29.9	26,653	42.4
*H. salicifolia*		MT512455	155,315	36.7	83,137	34.6	18,866	29.9	26,656	42.4
		MW392804	155,420	36.7	83,249	34.6	18,859	30.0	26,656	42.4

GC, guanine–cytosine; LSC, large single-copy; SSC, small single-copy; IR, inverted repeat.

The LSC regions ranged from 83,022 bp (*H. gyantsensis*, NC_044478) to 84,072 bp (*H. rhamnoides* subsp. *yunnanensis*, NC_044479), and the SSC regions from 18,586 bp (*H. neurocarpa*, MW791512) to 19,047 bp (*H. rhamnoides* subsp. *yunnanensis*). The IR regions ranged from 26,528 bp (*H. neurocarpa* subsp. *neurocarpa*, MT512453, and *H. neurocarpa*, MW791512) to 26,672 bp (*H. gyantsensis*). Guanine–cytosine (GC) contents were highly similar across species (36.6–36.7% for the whole genome, 34.5–34.6% for the LSC, 29.8–30.0% for the SSC, and 42.4% consistently for the IRs; Table 1).

Reannotation of all plastid genome sequences, excluding that determined in this study, identified 131 functional genes comprising 85 PCGs, 38 transfer RNA (tRNA), and eight ribosomal RNA (rRNA) genes (Table S5). Of these, four rRNA, eight tRNA, and seven PCGs were duplicated in both IR regions; 13 PCGs were located in the SSC region; and the remaining genes were located in the LSC region (Fig. 1). The pseudogenized *infA* was excluded from the list of functional genes.

Fourteen genes contained a single intron, including nine PCGs (*petB*, *petD*, *atpF*, *rps16*, *rpl2*, *rpl16*, *rpoC1*, *ndhA*, and *ndhB*) and five tRNA genes (*trnK-UUU*, *trnL-UAA*, *trnV-UAC*, *trnI-GAU*, and *trnA-UGC*). Three PCGs (*rps12*, *clpP*, and *ycf3*) contained two introns each. Of the 17 intron-containing genes, *ndhA* was located in the SSC region; *trnA-UGC*, *trnI-GAU*, *rps12*, *rpl2*, and *ndhB* were located in the IR regions; and the remaining 11 genes were located in the LSC region.

### Codon usage bias

Codon usage frequency was determined in a total of 78 PCGs. All 64 codons, including the three termination codons (UGA, UAG, and UAA), were identified in the plastid genomes of all *Hippophae* accessions. Of these, leucine and cysteine were the most and least abundant amino acids, respectively (Table S6).

**Fig. 2:**
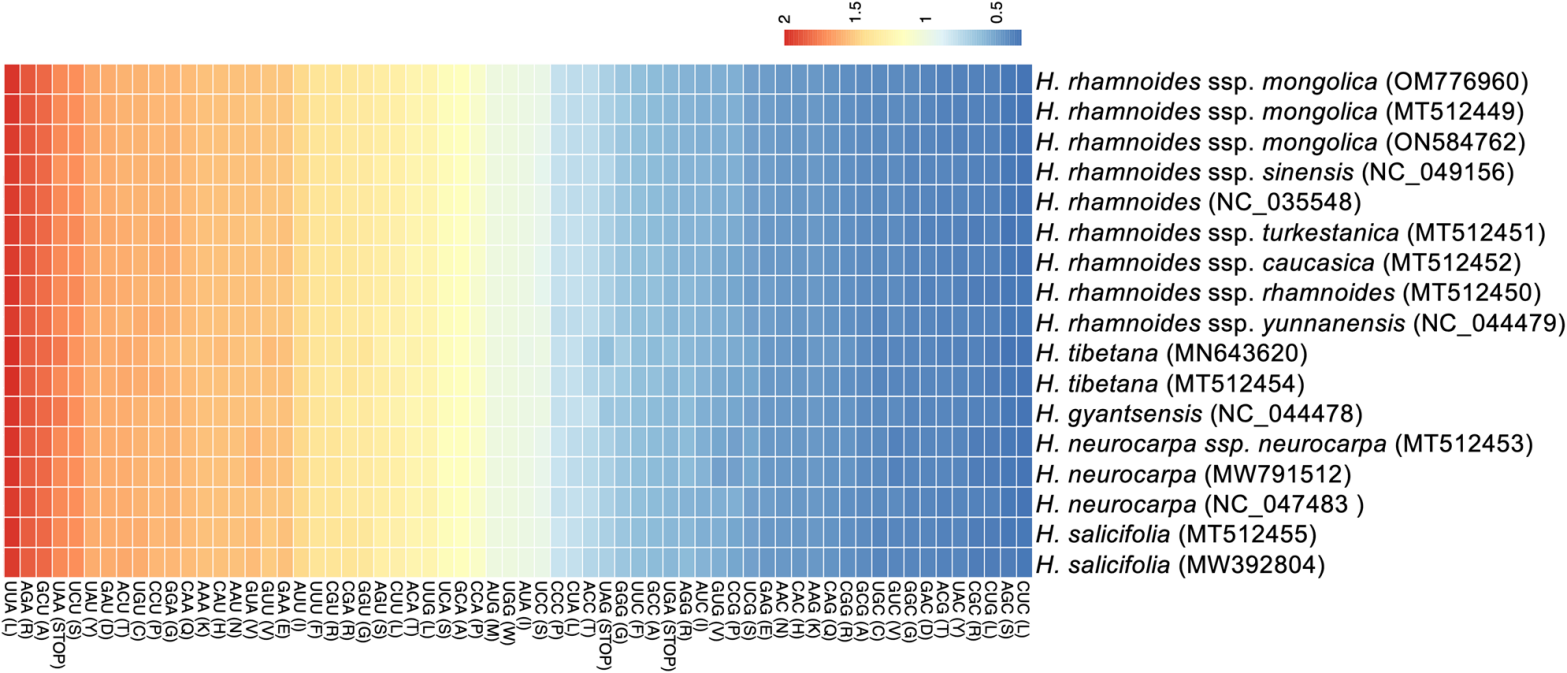
Heatmap of relative synonymous codon usage in *Hippophae*. Higher and lower RSCU values are indicated in red and blue, respectively.

RSCU analysis showed that, across all *Hippophae* accessions, nearly every amino acid with synonymous codons displayed a similar usage bias (Fig. 2). The codon with the highest RSCU value (1.96–2.00) was UUA (encoding leucine), whereas the lowest (0.32–0.34) was CUC (also encoding leucine), indicating a strong codon usage bias. Of the 64 codons, 30 had RSCU values greater than 1; all of these, except for UUG (encoding leucine), ended with A or U (Fig. 2, Table S6). In contrast, 32 codons had RSCU values below 1, 29 of which ended with G or C.

### Phylogenetic relationships

**Fig. 3:**
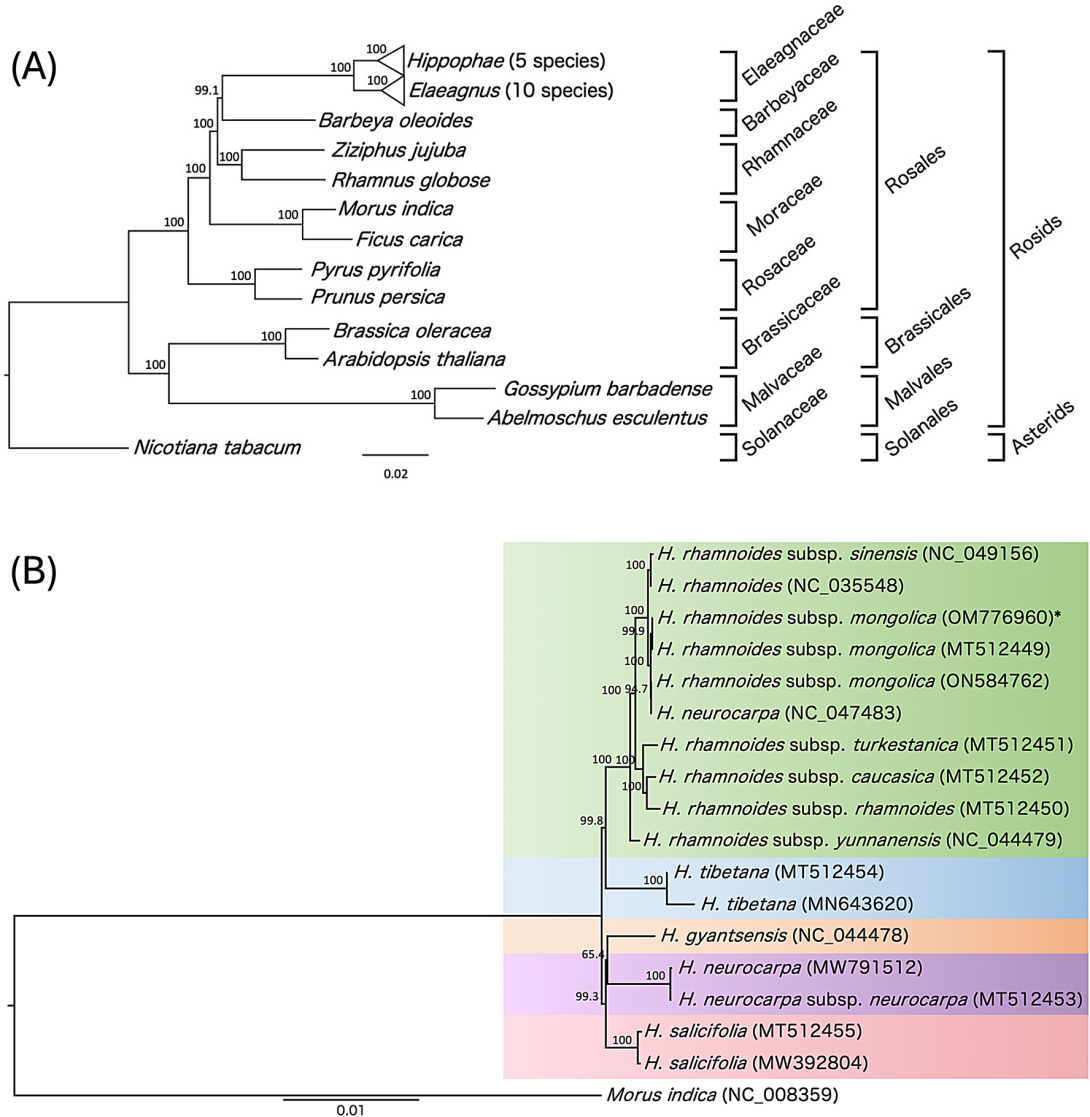
ML phylogenetic trees depicting evolutionary relationships among rosid species and accessions of *Hippophae*, inferred from plastid PCGs. (**A**) ML tree reconstructed using concatenated dataset of 78 plastid PCGs from 26 representative rosid taxa, including *Hippophae* and *Elaeagnus* species, with *N. tabacum* designated as the outgroup. (**B**) ML tree focusing on 17 plastid genome accessions representing five *Hippophae* species, with *M. indica* (NC_008359) used as the outgroup. One accession of *H. neurocarpa* (NC_047483) is nested within the *H. rhamnoides* clade, suggesting a potential hybrid origin involving subsp. *sinensis*. Bootstrap support values are provided at each node. * The sequence was newly determined in this study.

ML-based phylogenetic analysis using a nucleotide sequence matrix of 78 PCGs [from 26 rosid species including five *Hippophae* species (17 accessions) and 10 *Elaeagnus* species (16 accessions)] indicated well-resolved phylogenetic relationships with high bootstrap support, consistent with current taxonomic classification within the rosids (Fig. 3A). The monophyly of *Hippophae* was confirmed.

Within *Hippophae*, *H. rhamnoides*, *H. tibetana*, *H. gyantsensis*, *H. neurocarpa*, and *H. salicifolia* formed distinct clades (Fig. 3B), further confirming the monophyly of *H. rhamnoides*. One accession of *H. neurocarpa* (NC_047483) was nested within the *H. rhamnoides* clade, consistent with previous reports^58^, which suggested that it represents a hybrid between *H. rhamnoides* subsp. *sinensis* and *H. neurocarpa*. Subspecies of *H. rhamnoides* were grouped into three clusters: (i) *sinensis* (Asian) and *mongolica* (Asian); (ii) *yunnanensis* (Asian); and (iii) *turkestanica* (Central Asian), *caucasica* (Asia Minor), and *rhamnoides* (European).

### Characteristics of SSRs

**Fig. 4:**
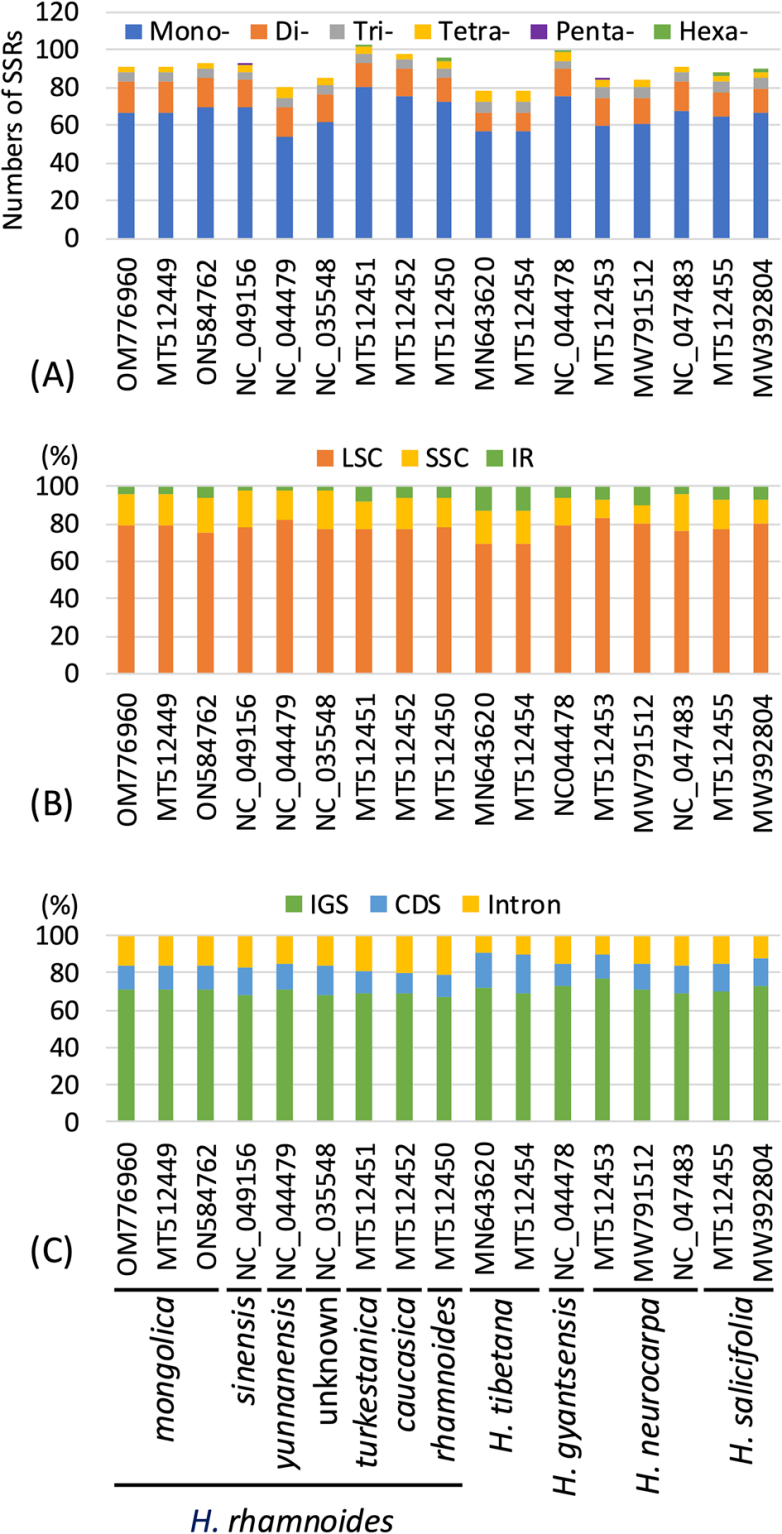
Comparative analysis of SSRs in plastid genomes of *Hippophae* species. **(A)** Numbers of SSRs categorized by motif length. **(B)** Distribution of SSRs across plastid genome regions: LSC, SSC, and IR. **(C)** Distribution of SSRs among genomic feature categories: IGS, intron, and coding sequence (CDS). LSC, large single-copy region; SSC, small single-copy region; IR, inverted repeat; IGS, intergenic spacer.

We identified 78–103 SSRs in each *Hippophae* plastid genome (Fig. 4A, Table S7). The proportions of mono-, di-, tri-, tetra-, penta-, and hexanucleotide SSRs were 67.5–77.7%, 12.5–20.0%, 4.0–7.7%, 3.1–7.5%, 0–1.2%, and 0–2.3%, respectively (Fig. 4A, Table S7). These plastid genomes shared a high prevalence of A/T-rich SSRs (Table S7). In all accessions, the most frequent motif was poly(A/T), followed by AT/AT repeats. More SSR loci were located in the LSC region than the SSC and IR regions (Fig. 4B). *Hippophae tibetana* had more SSRs in the IR regions than any other species. Across all accessions, most SSRs were situated in intergenic spacer (IGS) regions (66.7–76.5%, Fig. 4C). In *H. rhamnoides*, *H. gyantsensis* and *H. neurocarpa*, introns contained the second largest number of SSRs, followed by coding sequences. In contrast, in *H. tibetana* and *H. salicifolia*, coding sequences ranked second, followed by introns.

### Variation in IR boundaries

**Fig. 5:**
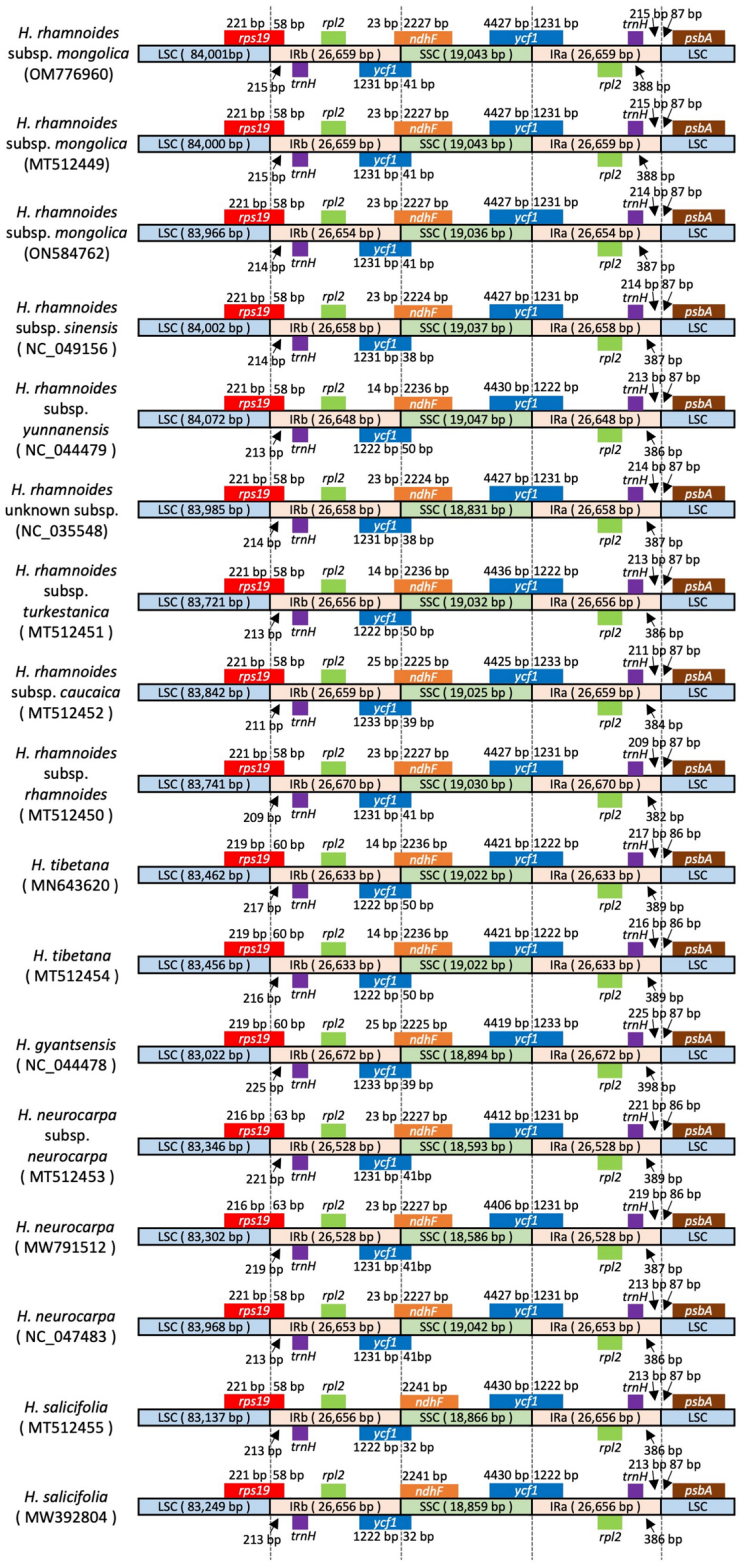
Comparison of IR boundary regions among *Hippophae* plastid genomes. The diagram shows the relative positions and lengths of genes adjacent to the LSC/IRb, IRb/SSC, SSC/IRa, and IRa/LSC junctions. Numbers indicate the distances (in base pairs) between the junctions and adjacent genes or lengths of gene segments extending into adjacent regions. LSC, large single-copy region; SSC, small single-copy region; IR, inverted repeat; IGS, intergenic spacer.

The expansion and contraction of the IR regions are important features of plastid genomes, contributing to variation in genome size and serving as useful markers for phylogenetic analysis and species/subspecies identification. Based on the reevaluated IR lengths of *H. tibetana* (MT512454), *H. gyantsensis* (NC_044478), *H. neurocarpa* (MT512453 and MW791512), and *H. salicifolia* (MW392804), we compared the IR boundaries among the 17 *Hippophae* plastid genomes (Fig. 5). Previous reports based on the original database annotation described *rps19* in *H. salicifolia* (MW392804, identical to NC_056188) as being separated from the LSC/IRb junction by 70 bp^41,42^. In contrast, our reevaluation placed the LSC/IRb boundary within *rps19*, consistent with the configuration observed in the other *Hippophae* accessions (Fig. 5).

Overall, the IR boundaries were generally conserved, with only slight variation in junction positions. The junctions were typically positioned as follows: LSC/IRb (within *rps19*), SSC/IRa (within *ycf1*), and IRa/LSC (between *trnH* and *psbA*). For example, the LSC/IRb boundary was consistently associated with *rps19* across accessions, with only minor shifts in junction positions, reflecting limited IR expansion/contraction in the genus (Fig. 5). The IRb/SSC junction showed clearer variation: in all *Hippophae* accessions except the two *H. salicifolia* accessions, it was located within *ndhF*; in *H. salicifolia*, the *ndhF* gene ended exactly at the IRb/SSC border (Fig. 5). The *trnH* gene was consistently located in the IR region and duplicated.

### Sequence divergence of plastid genome

**Fig. 6:**
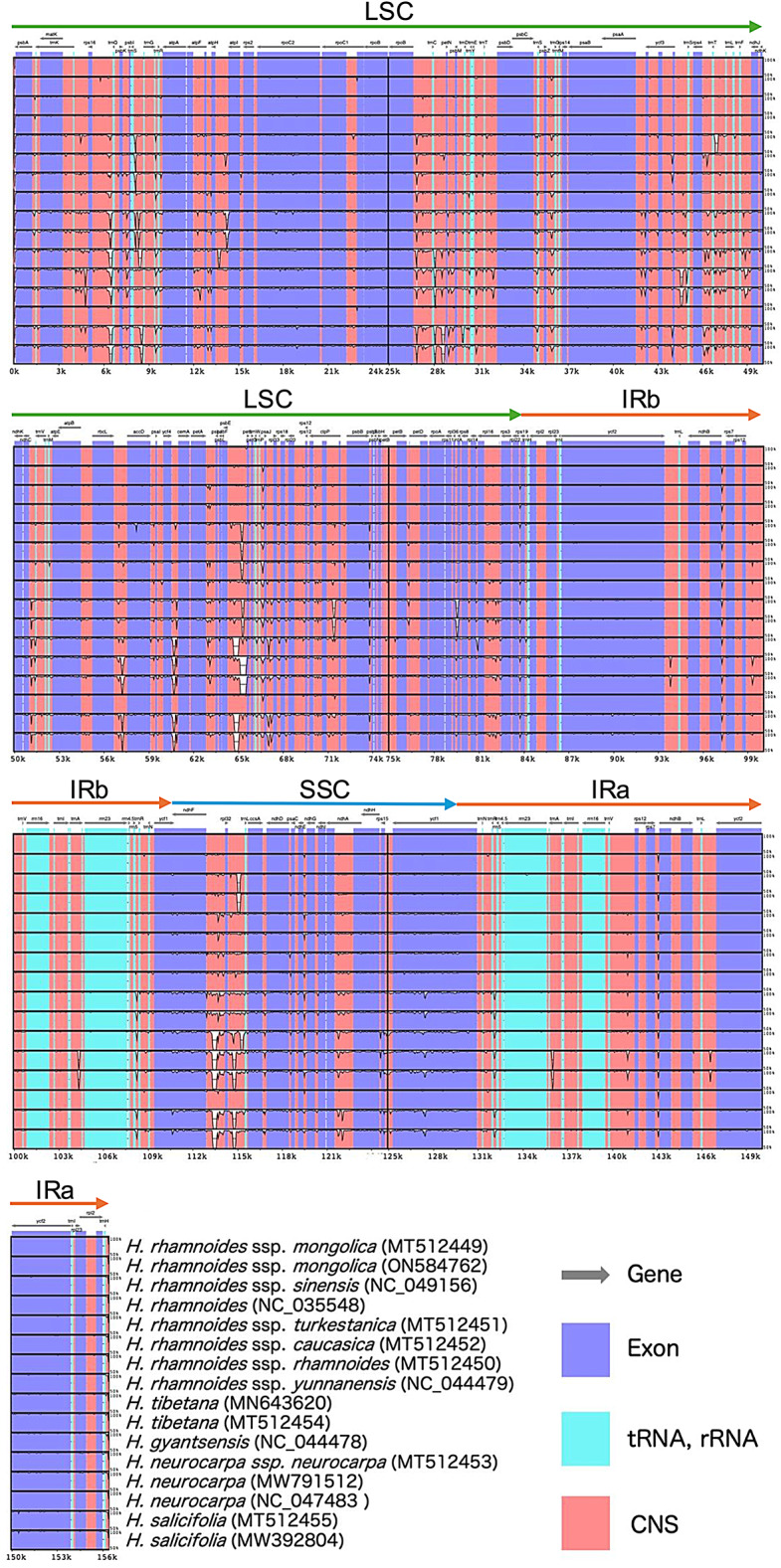
Comparative analysis of plastid genomes among 16 *Hippophae* accessions. The complete plastid genome sequences were aligned using mVISTA, with *H. rhamnoides* subsp. *mongolica* cv. Prevoskhodnaya as the reference. The analysis highlights conserved and variable regions among the accessions. Purple bars represent exons, sky-blue bars represent untranslated regions (tRNA and rRNA), and red bars represent conserved non-coding sequences (CNS). The scale on the right indicates the percentage identity between plastid genome sequences (50–100%).

We compared the complete plastid genome sequences of the 16 *Hippophae* accessions, with *H. rhamnoides* subsp. *mongolica* cv. Prevoskhodnaya as a reference (Fig. 6), and found that they were relatively conserved, with no genomic rearrangements, such as inversions or translocations. At the nucleotide sequence level, the LSC and SSC regions were more divergent than the two IR regions. The non-coding regions were clearly less conserved than the coding regions. Many sequence variations were identified in the non-coding regions. A total of 46 variable regions were detected, 39 of which were located in IGSs (*trnK–rps16*, *rps16–trnQ*, *psbK–psbI*, *trnS–trnG*, *trnG–trnR*, *trnR–atpA*, *atpH–atpI*, *rpoB–trnC*, *trnC–petN*, *petN–psbM*, *psbM–trnD*, *trnE–trnT*, *trnT–psbD*, *psbZ–trnG*, *psaA–ycf3*, *ycf3–trnS*, *rps4–trnT*, *trnT–trnL*, *trnF–ndhJ*, *ndhC–trnV*, *rbcL–accD*, *ycf4–cemA*, *petA–psbJ*, *psbE–petL*, *trnP–psaJ*, *psaJ–rpl33*, *rpl33–rps18*, *psbB–psbT*, *petB–petD*, *rpl36–infA*, *rpl14–rpl16*, *rpl22–rps19*, *ycf2–trnL*, *rps12–trnV*, *rrn5–trnR*, *ndhF–rpl32*, *rpl32–trnL*, *ccsA–ndhD*, and *ndhE–ndhG*) and six within introns of *atpF*, *ycf3*, *clpP*, *rpl16*, *trnA*, and *ndhA*. One variable region was located in the coding region of *ycf1*.

**Fig. 7:**
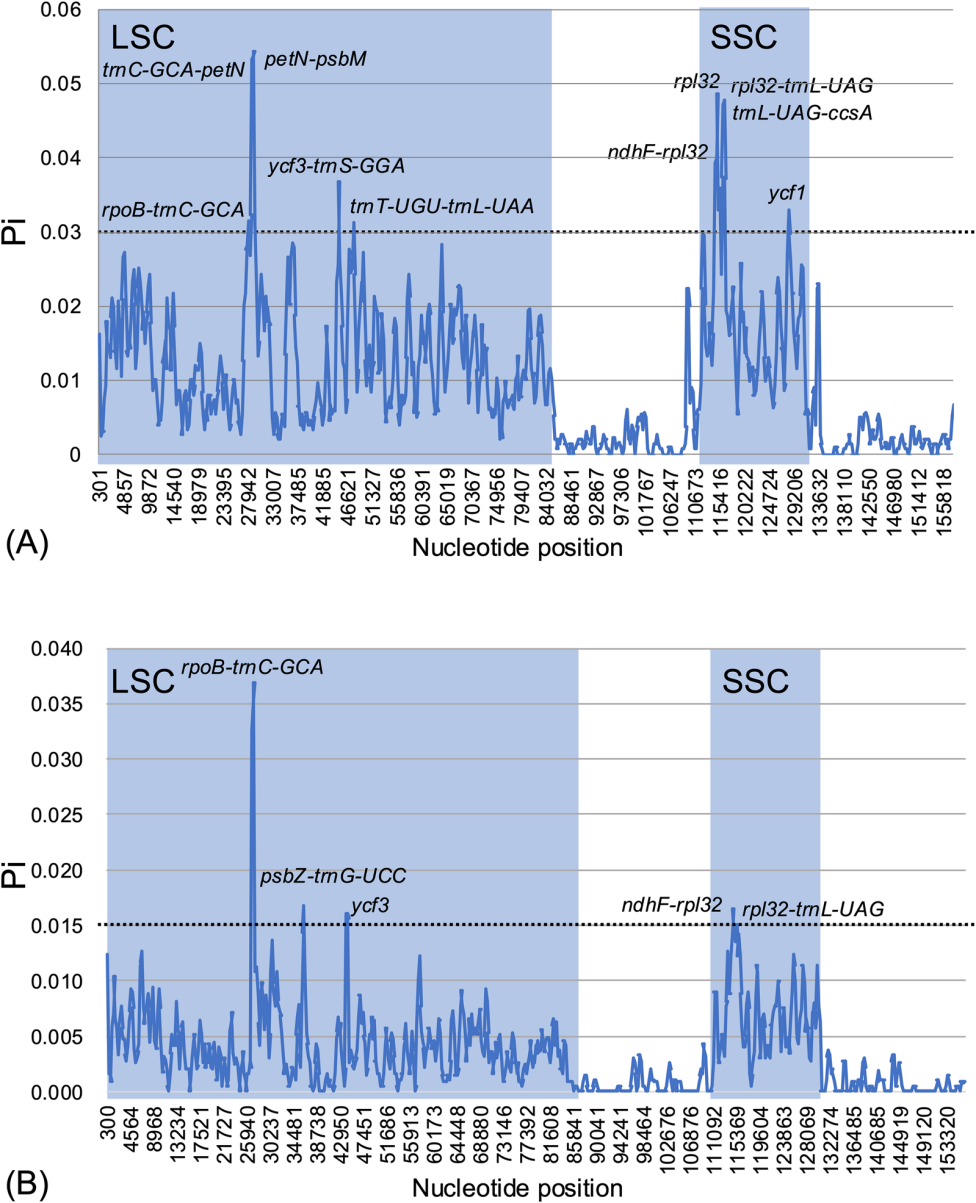
Nucleotide diversity (Pi) across plastid genomes of *Hippophae* based on sliding window analysis. Pi values were calculated using a window length of 600 bp and step size of 200 bp. (**A**) Interspecific nucleotide diversity among five *Hippophae* taxa (species/subspecies): *H. neurocarpa* subsp. *neurocarpa* (MT512453), *H. tibetana* (MT512454), *H. salicifolia* (MT512455), *H. rhamnoides* subsp. *mongolica* (OM776960), and *H. gyantsensis* (NC_044478). Highly variable regions with Pi values > 0.030 are indicated. (**B**) Intraspecific nucleotide diversity among *H. rhamnoides* subsp. *rhamnoides* (MT512450), *caucasica* (MT512452), *turkestanica* (MT512451), *yunnanensis* (NC_044479), *sinensis* (NC_049156), and *mongolica* (OM776960). Highly variable regions with Pi values > 0.015 are indicated.

The sliding window analyses of the interspecific and intraspecific nucleotide diversity (Pi) within *Hippophae* and *H. rhamnoides*, respectively, returned relatively low values (0–0.054, average of 0.0099 for *Hippophae*, Fig. 7A; 0–0.037, average of 0.0035 for *H. rhamnoides*, Fig. 7B). The IR regions exhibited lower divergence with smaller Pi-values than the SSC and LSC regions. Non-coding regions were generally more variable than coding regions. Ten highly variable interspecific regions (Pi > 0.030) were identified within *Hippophae*, including eight IGS regions (*rpoB–trnC–GCA*, *trnC–GCA–petN*, *petN–psbM*, *ycf3–trnS–GGA*, *trnT–UGU–trnL–UAA*, *ndhF–rpl32*, *rpl32–trnL–UAG*, and *trnL–UAG–ccsA*) and two coding regions (*rpl32* and *ycf1*). Within *H. rhamnoides*, six intraspecific divergence hotspots (Pi > 0.015) were identified: four IGS regions (*rpoB–trnC–GCA*, *psbZ–trnG–UCC*, *ndhF–rpl32*, and *rpl32–trnL–UAG*) and two Pi peaks within the second intron of *ycf3*. Three regions (*rpoB–trnC–GCA*, *ndhF–rpl32*, and *rpl32–trnL–UAG*) were consistently identified as hypervariable in both the interspecific and intraspecific analyses.

## Discussion

In this study, the plastid genomes of 17 *Hippophae* accessions (five species) demonstrated a typical quadripartite structure comprising a LSC, SSC, and two IR regions (154,944–156,415 bp in length). Reevaluation of gene annotations indicated that each plastid genome was annotated with 85 PCGs, 38 tRNAs, and eight rRNAs, with a duplication of four rRNA, eight tRNA, and seven PCGs (Table S5), consistent with previous findings on *Hippophae*^41,42,58,59^. The genes were also assigned in the same order (Fig. 1). These results showed that plastid genome structures were highly conserved in *Hippophae.* A discrepancy was noted for the presence of *matK*. While a previous plastid genome study reported an apparent absence of *matK* in *H. rhamnoides* subsp. *mongolica*^42^, we reannotated the GenBank-deposited plastid genome of subsp. *mongolica* (OR438663) using GeSeq and recovered an intact *matK* annotation. The *matK* nucleotide sequence (nucleotide positions 1715–3232 in OR438663) was identical to that of subsp. *mongolica* cv. Prevoskhodnaya (OM776960) analyzed in this study, as well as two additional GenBank-deposited subsp. *mongolica* plastid genomes (MT512449 and ON584762), suggesting that the previously reported absence is likely attributable to annotation/reporting differences rather than true gene loss.

The 64 codons in the plastid genomes showed essentially similar usage bias across *Hippophae* accessions (Fig. 2). We identified 30 highly preferred codons (RSCU > 1.0), two codons showing no bias (RSCU = 1.0), and 32 less-preferred codons (RSCU < 1.0). In *Hippophae*, preferred codons largely ended in A or U. UUA (Leu) was the most highly favored codon. However, UUG (Leu) was also relatively preferred despite being G-ending. This pattern is consistent with the general preference for A/U-ending codons widely reported in plastid genes across diverse plant lineages, reflecting underlying nucleotide compositional tendencies^60^. A similar preference for UUG has also been reported in the closely related genus *Elaeagnus* (Elaeagnaceae)^61^. In contrast, rosalean lineages outside Elaeagnaceae, such as Rhamnaceae (*Rhamnus* and *Frangula*), exhibit markedly different leucine codon usage, in which UUA is underrepresented (RSCU < 1.0) while UUG is strongly favored (RSCU ≥ 1.94)^62^. This clear contrast highlights pronounced lineage-dependent variation in plastid genome codon preference within Rosales. Nevertheless, minor shifts in RSCU values among *Hippophae* species were observed (Table S6), suggesting subtle lineage-dependent compositional biases. For example, the RSCU of UUA (Leu) was slightly higher in *H. tibetana* (2.00) than in most *H. rhamnoides* accessions (1.96–1.97) (Table S6).

The ML phylogenetic analyses confirmed the monophyly of the genus and demonstrated clear delimitations between the species and subspecies (Fig. 3), underscoring the relevance of plastid genomes for evolutionary studies in *Hippophae*. These results also highlight the potential of plastid genomes in the development of DNA markers for distinguishing *Hippophae* species and *H. rhamnoides* subspecies. The analyses indicated an issue regarding the systematic delineation of *H. tibetana*, which warrants further investigation. Previous phylogenetic analyses based on nuclear ITS sequences placed *H. tibetana* near the base of the genus^63^, and DNA barcoding analyses based on the ITS2 region likewise supported this basal placement^30^. In contrast, plastid barcoding using the *psbA–trnH* region positioned *H. tibetana* close to *H. rhamnoides* within the same major clade^30^. Moreover, phylogenetic reconstruction using five chloroplast loci grouped *H. tibetana* and *H. rhamnoides* into the same clade, suggesting a closer relationship^64^. This discordance likely reflects differences in the evolutionary histories captured by nuclear (ITS) versus plastid markers, as well as the limited number of loci in earlier studies. Such cytonuclear discordance between plastid and nuclear phylogenies has been widely reported in angiosperms, particularly at shallow taxonomic levels^65^. This incongruence may reflect biological processes such as introgressive hybridization and chloroplast capture, which can distort phylogenetic relationships inferred from plastid genome data^66^. In addition, incomplete lineage sorting of nuclear loci may also contribute to discordant signals^67^. Therefore, although plastid phylogenies provide valuable insights into relationships within *Hippophae*, additional nuclear genomic data will be necessary to further evaluate potential reticulate evolutionary histories in this genus. In the present analysis of 78 plastid PCGs, *H. tibetana* clustered with *H. rhamnoides*, consistent with recent plastid genome-wide analyses^41,42^. To resolve discrepancies among studies and provide a more comprehensive reconstruction of the evolutionary history of *H. tibetana*, integrative phylogenomic approaches incorporating both nuclear and plastid datasets, such as RAD-seq^68^ and Hyb-seq^69^, will be required. Our dataset expands taxon sampling across the genus compared with previous plastid genome studies, which were generally based on relatively limited species and/or subspecies sampling. By analyzing 17 accessions representing five *Hippophae* species, we were able to (i) directly compare interspecific plastid genome variation, (ii) confirm the placement of *H. tibetana* within the *H. rhamnoides* clade using 78 PCGs, and (iii) detect lineage- and species-specific genomic patterns, including differences in SSR distribution and IR boundary configurations, which cannot be evaluated under a limited taxon-sampling framework.

The SSR landscape observed in the present study is broadly consistent with previous plastid genome studies of *Hippophae*^41,42^, showing a predominance of A/T-rich mononucleotide repeats, with SSRs preferentially located in intergenic regions. In our dataset of 17 accessions representing five *Hippophae* species, each plastid genome contained 78–103 SSRs, of which 67.5–77.7% were mononucleotide SSRs, and the most frequent motif was poly(A/T), followed by AT/AT repeats. Our expanded sampling further revealed lineage-level variation in SSR distribution, most notably the relatively higher number of SSR loci located in IR regions in *H. tibetana* (Fig. 4B), a pattern that was not highlighted in previous studies and may provide additional taxon-informative signals for marker development. Minor differences in total SSR counts among studies may reflect differences in SSR mining tools and parameter settings; nevertheless, the major SSR landscape is highly concordant. Since their usefulness as polymorphic markers for chloroplast genomes was first demonstrated in plant population genetics^70^, chloroplast genome SSRs have been widely used as valuable DNA markers in many plant species, including wheat^71^ and pear^72^, providing insights into genetic variation and evolutionary relationships. Given that 66.7–76.5% of the SSR loci were located in IGS regions (Fig. 4C), where selective constraints are generally weaker and mutation rates tend to be higher than in coding regions, we anticipate substantial variation in the number of repeats at each SSR locus. Thus, plastid genome SSRs may be especially valuable in population genetics, evolutionary, and conservation studies of *Hippophae* species and *H. rhamnoides* subspecies.

In plastid genomes, the nucleotide substitution rates at both synonymous sites and in noncoding regions are markedly lower in the IR than in the single-copy regions^73–75^. This is attributed primarily to the presence of two nearly identical IR copies that facilitate frequent homologous recombination, particularly through gene conversion^74,75^. This process corrects mismatches between IR copies, effectively repairing point mutations and maintaining sequence identity. The size of the IR region varies considerably among plant lineages. Evolutionary studies indicate frequent expansion and contraction events at the IR boundaries^39,76^, which typically occur through the inclusion or exclusion of adjacent genes from the LSC or SSC regions by homologous recombination or double-strand break repair^74,77,78^. The reevaluation of IR boundaries represents an annotation-level refinement based on pairwise alignment of sequences flanking the IR/SC junctions, rather than experimental correction of the original plastid genome sequences. In the current study, the expansion and contraction of the IR regions showed similar tendencies across *Hippophae* (Fig. 5). Variation at the LSC/IRb junction was mainly associated with the position of *rps19* relative to the IR boundary (Fig. 5), indicating minor lineage-dependent shifts in IR expansion/contraction among *Hippophae* accessions. We confirmed the complete duplication of *trnH* in *Hippophae* (Fig. 5), a feature that was first reported among rosids at the LSC/IRb and LSC/IRa border regions in *Elaeagnus*^39^. This duplication clearly occurred before the divergence of *Elaeagnus* and *Hippophae*. In our dataset of 17 plastid genomes, only *H. salicifolia* exhibited an IRb/SSC boundary configuration in which the *ndhF* gene terminates at the junction, a feature that may be useful for developing a species-specific molecular marker. Although similar IRb/SSC boundary configurations involving *ndhF* have been illustrated in previous studies^41^, the configuration reported there does not match our consistently reannotated IR boundaries, likely reflecting annotation differences, whereas our results are consistent with^42^.

The highly polymorphic regions in plant plastid genomes provide a promising basis for developing molecular markers for phylogenetic analysis. Several plastid genome-based markers have been developed in diverse plant groups, including *Siraitia*^79^, *Gleditsia*^80^, *Taxodium* hybrids^81^, and Polygonoideae^82^. In *Hippophae*, plastid genome structures were generally conserved, with coding regions highly conserved and non-coding loci more variable (Fig. 6). Using mVISTA, we identified 46 variable regions, of which 45 were located in non-coding regions and only one in a coding region. These non-coding regions are generally less constrained and thus more informative for marker development.

Previous plastid genome studies of *Hippophae* conducted interspecific comparisons at the genus level^41^ or focused primarily on subspecies-level sampling within *H. rhamnoides*^42^. In contrast, our study expanded taxon sampling across multiple species and subspecies, enabling a more comprehensive detection of variable regions across the genus. Because variation occurred both among species and among subspecies, nucleotide diversity (Pi) analyses were conducted separately for interspecific comparisons among five *Hippophae* taxa (species/subspecies) and for intraspecific comparisons among six subspecies of *H. rhamnoides* (Fig. 7). Sliding-window analyses in DnaSP identified 10 interspecific variable regions (eight noncoding and two coding) and six intraspecific variable regions (all noncoding, including four IGSs and two hotspots within the second intron of *ycf3*). For genus-wide species discrimination (interspecific comparison), eight highly variable IGS regions (*rpoB–trnC–GCA*, *trnC–GCA–petN*, *petN–psbM*, *ycf3–trnS–GGA*, *trnT–UGU–trnL–UAA*, *ndhF–rpl32*, *rpl32–trnL–UAG*, and *trnL–UAG–ccsA*) and two coding regions (*rpl32* and *ycf1*) showed high Pi values (Pi > 0.030; Fig. 7A). Within *H. rhamnoides* (intraspecific comparison), six divergence hotspots were detected (Pi > 0.015; Fig. 7B), including four IGS regions (*rpoB–trnC–GCA*, *psbZ–trnG–UCC*, *ndhF–rpl32*, and *rpl32–trnL–UAG*) and two hotspots within the second intron of *ycf3*.

Overall, both genome-wide mVISTA and Pi-based analyses indicated that sequence divergence is largely concentrated in noncoding regions. In particular, three regions (*rpoB–trnC–GCA*, *ndhF–rpl32*, and *rpl32–trnL–UAG*) were consistently identified as highly variable in both mVISTA and nucleotide diversity (Pi) analyses across interspecific and intraspecific comparisons, making them high-priority candidates for diagnostic plastid markers for species and subspecies delimitation. Several major hotspots identified in our interspecific Pi analysis overlap with those reported in previous genus-wide *Hippophae* plastid genome studies^41^, supporting the robustness of these loci across sampling schemes. In contrast, overlap with the subspecies-focused analysis within *H. rhamnoides*^42^ is more limited, likely reflecting differences in taxon scope and sampling design. Notably, previous studies either calculated nucleotide diversity using mixed interspecific and intraspecific datasets^41^ or focused primarily on subspecies-level sampling^42^, whereas our study expanded taxon sampling across multiple species and subspecies and separated interspecific from intraspecific Pi analyses, allowing clearer assessment of plastid genome divergence patterns.

In conclusion, this study provides a comprehensive classification of *Hippophae* based on complete plastid genomes. The genomes exhibited a conserved quadripartite structure and gene order across species. Reannotation and reevaluation of the IR boundaries clarified several database inconsistencies. Phylogenomic analyses of the 78 PCGs confirmed the monophyly of *Hippophae* (and *H. rhamnoides*) and placed *H. tibetana* in a clade with *H. rhamnoides*, highlighting a key issue for future systematic work. Abundant A/T-rich SSRs and 46 variable regions were identified as promising candidates for developing molecular markers, with interspecific and intraspecific hotspots shared across taxa. IR boundary patterns were generally stable, including *trnH* duplication across the genus and the distinct *ndhF*–IRb/SSC boundary configuration in *H. salicifolia*. Collectively, these features provide candidate loci for developing diagnostic DNA markers, which require further population-level validation and may support breeding, conservation, and resource management. Future work should expand sampling across *H. rhamnoides* and other *Hippophae* species and validate candidate markers at the population scale. Integrating high-resolution nuclear datasets (e.g., RAD-seq/Hyb-seq) will further refine phylogenetic relationships—particularly those involving *H. tibetana*—and complement plastid-based inferences by elucidating links between plastid variation and ecological or geographic diversification.

## Supplementary Information

Below is the link to the electronic supplementary material.

Supplementary Material 1

## Data Availability

The complete plastid genome assemblies and annotations generated in this study are available in the NCBI GenBank database (*Hippophae rhamnoides* subsp. *mongolica* cv. Prevoskhodnaya: GenBank accession OM776960).
